# COVID-19 Fear Among Pakistanis: Psychometric Evaluation of the Fear of COVID-19 Scale Using Item Response Theory and Confirmatory Factor Analysis

**DOI:** 10.1007/s11469-021-00656-2

**Published:** 2021-11-29

**Authors:** Irfan Ullah, Muhammad Junaid Tahir, Sajjad Ali, Rabia Waseem, Mark D. Griffiths, Mohammed A. Mamun, Chung-Ying Lin, Amir H. Pakpour

**Affiliations:** 1 Kabir Medical College, Gandhara University, Peshawar, Pakistan; 2 CHINTA Research Bangladesh, Dhaka, Bangladesh; 3 Ameer-Ud-Din Medical College, Lahore, Pakistan; 4 Ziauddin University, Karachi, Pakistan; 5 Karachi Medical and Dental College, Karachi, Pakistan; 6 International Gaming Research Unit, Psychology Department, Nottingham Trent University, Nottingham, UK; 7 Department of Public Health and Informatics, Jahangirnagar University, Dhaka, Bangladesh; 8 Institute of Allied Health Sciences, College of Medicine, National Cheng Kung University, Tainan, Taiwan; 9 Department of Nursing, School of Health and Welfare, Jönköping University, Jönköping, Sweden

**Keywords:** COVID-19, Fear of COVID-19 Scale, Fear, FCV-19S Urdu validation, Pakistani population

## Abstract

The Fear of COVID-19 Scale (FCV-19S) assesses the fear of the novel coronavirus disease 2019 (COVID-19) and has been translated and validated into over 20 languages. The present study conducted confirmatory factor analysis (CFA) and item response theory (IRT) analyses on the FCV-19S among a sample of 937 Pakistani adults (mean [SD] age of 25.83 [11.80] years; 537 [57.3%] females). The CFA and IRT confirmed the unidimensionality of the FCV-19S. The Likert-type scale used in the FCV-19S was supported by the proper threshold orderings. Additionally, no DIF contrast had an absolute value larger than 0.5 regarding the participants’ characteristics of gender, age, living status, and education in the IRT findings. The FCV-19S was found to be valid and reliable with strong psychometric properties among the Pakistani adult population.

The novel coronavirus disease-2019 (COVID-19) outbreak, caused by Severe Acute Respiratory Syndrome Coronavirus-2 (SARS-CoV-2), first originated in Wuhan in the Hubei province China (Lipsitch et al., 2020). SARS-CoV-2 is a strain of coronavirus that belongs to the same family of viruses which accounted for both SARS (Severe Acute Respiratory Syndrome) outbreak in 2003 and MERS (Middle East Respiratory Syndrome) outbreak in 2012 (Mukhtar & Mukhtar, 2020). COVID-19 has become a global issue because of its high transmission and infection rates (Usman et al., 2020) and was declared as a global pandemic on March 11, 2020, by the World Health Organization (World Health Organization 2020). At the time of writing (November 2021), there were over 259.41 million COVID-19 confirmed cases reported worldwide and over 5.18 million deaths from the disease (Worldometer, 2021). In Pakistan (where the present study was carried out), there have been over 1.28 million cases and over 28,670 deaths since February 26, 2020 (Ali et al., 2020; Worldometer, 2021).

Because of relatively high infection and mortality rates along with the lack of proven clinical treatments, individuals are afraid and worried about becoming infected with COVID-19 (Ashraf et al., 2021; Kobayashi et al., 2020; Lin, 2020; Mamun & Griffiths, 2020a; Rajabimajd et al., 2021). Fear is a co-morbid characteristic of an infectious disease compared with other conditions and is usually elevated when the infection transmits rapidly and invisibly, accounting for major morbidity and mortality (Pappas et al., 2009; Rajabimajd et al., 2021). A recent study reported evidence of increasing fear levels due to COVID-19 pandemic across the world (Knipe et al., 2020), similar to past viral epidemics (e.g., SARS [Reynolds et al., 2008]; MERS [Bukhari et al., 2016]). Additionally, social marginalization and stigmatization derived from fear may cause individuals to deliberately deny they have early symptoms of the disease which are clinically important and may lead to increased number of undetected cases in the community during a disease outbreak (Sakib et al., 2020).

The COVID-19 pandemic has exaggerated fears globally and has led to stigma in some cases, and unfortunately, this fear may heighten the damage of the disease itself (Ahorsu et al., 2020; Pakpour & Griffiths, 2020). For instance, excessive fear of COVID-19 can lead to worsening of anxiety symptoms among individuals with pre-existing psychological issues and can facilitate psychological distress among those in the general population. In extreme cases, such psychological distress can lead to suicidal behaviors (Mamun & Griffiths, 2020b; Mamun & Griffiths 2020c). There is now growing evidence that fear of COVID-19 together with other psychological mechanisms (e.g., causes of severe stress and hardship from the economic decline during the pandemic) have led to suicides around the world including Pakistan and neighboring countries such as Bangladesh and India (e.g., Bhuiyan et al., 2020; Dsouza et al., 2020; Goyal et al., 2020; Griffiths & Mamun, 2020; Mamun & Griffiths, 2020a; Mamun & Ullah, 2020). In contrast, when fear is coupled with high efficacy and perceived benefits, it could act as a motivator for behavioral change related to COVID-19 (Lin et al., 2021). For instance, it has been shown that fear of COVID-19 helps in increased preventive behaviors such as spatial distancing and hand hygiene, indicating that fear has important role in the compliance with public health measures (Alyami et al., 2020; Winter et al., 2020).

Due to the unprecedented and ongoing nature of the COVID-19 pandemic and its’ increased psychological impact on individuals, developing brief and valid instruments to assess mental health suffering and consequences are highly needed. Consequently, Ahorsu et al. (2020) recently developed the Fear of COVID-19 Scale (FCV-19S) to help enrich global knowledge by assessing COVID-19 fear (and its corollaries and consequences) in different countries. The FCV-19S is a seven-item scale that is quick and easy-to-use and has shown very good psychometric properties in various cultural adaptation across the world including Arabic (Alyami et al., 2020), Bangla (Sakib et al., 2020), English (Perz et al., 2020; Winter et al., 2020), Greek (Tsipropoulou et al., 2020), Hebrew (Bitan et al., 2020), Italian (Soraci et al., 2020), Persian (Ahorsu et al., 2020), Russian (Reznik et al., 2020), Japanese (Masuyama et al., 2020), Chinese (Chang et al., 2020; Pakpour et al., 2020), Spanish (Broche-Pérez et al., 2020; Huarcaya-Victoria et al., 2020), Turkish (Haktanir, et l., 2020; Satici et al., 2020), Norwegian (Iversen et al., 2021), Portuguese (Soares et al., 2021), and Vietnamese (Nguyen et al., 2020). However, to the best of the present authors’ knowledge, the psychometric testing for the Urdu FCV-19S is insufficient. More specifically, the Urdu FCV-19S has only been tested in one study, which only used classical test theory (CTT) (Mahmood et al., 2020). Therefore, using different methods of psychometric testing (i.e., item response theory [IRT] and classical test theory [CTT]) to examine the Urdu FCV-19S is necessary to provide more psychometric evidence of the Urdu FCV-19S.

CTT is the most commonly used method to examine the psychometric properties of an instrument. Although CTT is widely accepted and used in psychometric studies, IRT (including Rasch analysis) is an alternative to evaluating an instrument’s psychometric properties and for estimating a participant’s underlying ability and the difficulty of each item. The advantages of using it include (i) separately evaluating the ability of the individual and the difficulty of the item; (ii) determining how different groups perceive the same item in different ways; (iii) checking the validity of the item and the unidimensionality of the entire instrument; and (iv) examining the appropriateness of the descriptors for the response (Lin & Pakpour, 2017). Therefore, the present study aimed to translate and validate the FCV-19S into Urdu language and to assess its psychometric properties using both CTT and IRT methods. More specifically, the primary research question for the present study was whether the Urdu FCV-19S had satisfactory psychometric properties in both CTT and IRT methods.

## Methods

### Participants and procedures

A cross-sectional online survey study was conducted from May 5 to 28 (2020) in Pakistan, targeting its general population. The survey was planned to recruit 900 participants, and when the target number was reached, the data collection ended (i.e., May 28, 2020). A convenience sampling technique was utilized to recruit participants. The online survey was hosted on *Google Forms* and was created and shared on popular Pakistani online platforms (e.g., *Facebook, WhatsApp,* etc.). Participants were also invited to share the online survey among their peers to achieve widespread response across the country. No missing values were found in the data file because the survey could only be submitted if all items were responded to. There was not a single response that needed deletion. Furthermore, no duplication occurred because all participants completed the survey only once. The inclusion criteria for participation were being (i) a Pakistani national residing in their homeland, (ii) at least 18 years of age, and (iii) able to speak Urdu as their first language. The final sample comprised 937 participants. A sample size of over 200 was adequate for the statistical analyses used in the present study (i.e., confirmatory factor analysis; Su et al. 2014). Additionally, sample size calculation suggested the size should be 860 using the following guidelines: type I error at 0.01, statistical power at 0.9, a null root mean square approximation error (RMSEA) at 0, and an alternative RMSEA at 0.05 (Preacher & Coffman, 2006). The study was approved by the University of Sargodha (Reference #SU/PSY/785-S) ethics committee and was carried out in accordance with human research ethics outlined in the Helsinki Declaration, 1975. The use of FCV-19S was granted in an email by the developers (i.e., Drs. Pakpour, Griffiths, and Lin). Informed consent was provided by all participants before completing the online survey. All participants were assured that their data would be anonymous and confidential.

### Urdu fear of COVID-19 scale

The fear of COVID-19 was assessed using the Urdu Fear of COVID-19 Scale, which was adapted from the original version (Ahorsu et al., 2020). The scale comprises seven items (e.g*.,* “I cannot sleep because I am worried about getting coronavirus-19”). The scoring is based on a 5-point Likert point response from 1 (*strongly disagree*) to 5 (*strongly agree*) with a range of 7–35. Higher scores indicate greater fear of COVID-19 (Ahorsu et al., 2020). The psychometric properties of the scale are presented in the “Results” section. The Urdu version has been found to be highly valid and reliable for using among general pupation in Pakistan (Mahmood et al., 2020). In the present study, the linguistic validity of the Urdu FCV-19S was confirmed using the following steps. First, bilingual experts in Urdu and English translated the English FCV-19S into Urdu. Second, the Urdu FCV-19S was then translated back to English by another bilingual speaker who was not aware of the original English FCV-19S. In both stages, two bilingual speakers who were fluent in English and Urdu checked all the translated versions and provided feedback to create a consensual version. Finally, a pilot study was carried out to check the readability and comprehension of the Urdu FCV-19S.

### Hospital Anxiety and Depression Scale (HADS)

The Urdu version of the HADS was used to assess anxiety and depression (Waqas et al., 2019). The scale comprises 14 items with seven items for depression (e.g., *“I still enjoy the things I used to enjoy”*) and seven for anxiety (e.g., *“I feel tense or wound up”*). Two of the items (i.e., Item 7 concerning appetite, and Item 10 concerning interest in things) are reverse scored. Each item is rated on 4-point Likert scale (0–3) with total scores ranging from 0 to 21. The HADS has been translated into Urdu and was found to be valid and reliable for using in Pakistan (Mumford et al., 1991). The Cronbach’s alpha for the Urdu HADS in the present study was 0.84 (anxiety subscale) and 0.73 (depression subscale); McDonald’s omega for the Urdu HADS in the present study was 0.84 (anxiety subscale) and 0.74 (depression subscale).

### Data analysis

The sample characteristics are reported using descriptive statistics. Cronbach’s alpha coefficient (α), McDonald’s omega coefficient (ω), inter-item correlations, and correlated item-total correlations were performed for internal consistency. A Cronbach’s alpha (α) and a McDonald’s omega (ω) of 0.70 or above indicates that reliability is acceptable (DeVellis, 2016; Nunnally & Bernstein, 1994). The impacts on the overall alpha correlation coefficient were evaluated in each item. The correlation of inter-item and correlations in item-total between 0.30 and 0.70 suggest medium to heavy inter-item associations (Ferketich, 1991). Both α and ω were performed using R software with the psych package (Revelle, 2021).

Three CFAs with the diagonally weighted least squares estimator were performed on the FCV-19S. CFA was performed using the R software with the lavaan package (Rosseel, 2012). The following criteria were used to support the fit of a single-factor structure: root mean square approximation error (RMSEA < 0.08); comparative fit index (CFI > 0.90); Tucker-Lewis index (TLI > 0.90); and standardized root mean square residual (SRMR < 0.08).

The basic aspects of the IRT parameters (including difficulty and discrimination) were tested using item characteristic curve (ICC) analysis using the partial credit model. Item fit in the IRT was assessed using the infit and outfit mean square (MnSq), and a MnSq between 0.5 and 1.5 indicates proper fit. Threshold ordering of the Likert-type scale of FCV-19S was tested with the following criteria: (i) average measure and step measure of the difficulties should be monotonically increased; (ii) infit and outfit MnSq should be between 0.5 and 1.5. Additionally, differential item functioning (DIF) of the FCV-19S items across subgroups (gender, age, living status, education status, residence status, and health status groups) was evaluated using the following criterion: absolute DIF contrast < 0.5. The IRT analyses were performed utilizing WINSTEPS software (version 4.3.0).

Moreover, concurrent validity of the FCV-19S was assessed using structural equation modeling (SEM) with the diagonally weighted least squares estimator between the FCV-19S, the anxiety subscale of the HADS, and the depression subscale of the HADS. In addition, the correlations between the latent FCV-19S score and the latent scores of HADS subscales were examined. The concurrent validity of the FCV-19S was assessed by comparing the correlations with the two HADS subscales with the use of SEM because SEM has the benefits of accounting for measurement errors occurring in the instruments (Zumbo, 2005). Moreover, the SEM model is supported when both RMSEA and SRMR < 0.08 and both CFI and TLI > 0.90. SEM were performed using R software with the lavaan package (Rosseel, 2012).

## Results

Table 1 presents the participants’ sociodemographic information. More specifically, the sample comprised 937 participants (mean age = 25.83 years [SD ± 11.80]; 57.3% females [*n* = 537]). Three-quarters of the participants were single (77.4%), almost all were Muslims (97.8%), two-thirds had an undergraduate education (63.7%), one-quarter were in full-time employment (24.4%), nine-tenths currently lived in urban areas (89.4%), and nine-tenths were non-smokers (87.8%). Cronbach’s alpha for the Urdu FCV-19S was 0.88 and McDonald’s omega for the Urdu FCV-19S was 0.88. CFA results indicated satisfactory psychometric properties for the single-factor structure of the FCV-19S (χ^2^ = 101.44 [df = 14]; *p* < 0.001; CFI = 0.980; TLI = 0.970; SRMR = 0.075), except for the unsatisfactory RMSEA with a slightly high value of 0.082. The factor loadings of the FCV-19S ranged between 0.67 and 0.79 (Table 2).

**Table 1:** Sociodemographic information of the present sample

Variable	N	%
Age *(mean and SD)*	25.83 ± 11.80	
Gender		
Male	400	42.7
Female	537	57.3
Marital status		
Single	725	77.4
Married	202	21.6
Divorced	5	0.5
Widow	5	0.5
Religion		
Islam	916	97.8
Hinduism	13	1.4
Others	8	0.8
Education		
No formal education	3	0.3
Primary	5	0.5
Secondary	44	4.7
Higher secondary	111	11.8
Undergraduate	597	63.7
Post-graduate	177	18.9
Employment		
Full-time employed	229	24.4
Part-time employed	60	6.4
Unemployed	94	10.0
Home maker	59	6.3
Full-time student	451	48.1
Part-time student	25	2.7
Others	19	2.1
Residence		
Rural	38	4.1
Urban	838	89.4
Semi-urban	61	6.5
Smoking		
Non-smoker	823	87.8
Current smoker	80	8.5
Former smoker	34	3.6
Self-reporting health status		
Very poor	4	0.4
Poor	22	2.3
Acceptable	229	24.4
Good	415	44.3
Very good	267	28.5

**Table 2:** Item difficulty and fit statistics for the Fear of COVID-19 Scale (FCV-19S)

Item	Mean (SD)	Skewness	Kurtosis	Factor loading	Discrimination	Difficulty	Infit MnSq	Outfit MnSq
I am most afraid of COVID-19	2.97 (1.05)	− 0.11	− 0.59	0.67	0.83	− 0.69	1.00	1.10
It makes me uncomfortable to think about COVID-19	2.99 (1.16)	− 0.22	− 0.97	0.73	1.11	-0.72	0.92	0.93
My hands become clammy when I think about COVID-19	2.07 (1.11)	0.94	0.19	0.69	0.96	0.97	1.02	1.01
I am afraid of losing my life because of COVID-19	2.67 (1.24)	0.15	− 1.14	0.74	1.01	− 0.16	1.04	1.02
When watching news and stories about COVID-19 on social media, I become nervous or anxious	3.11 (1.18)	− 0.33	− 0.91	0.71	0.98	− 0.93	1.04	1.03
I cannot sleep because I’m worrying about getting COVID-19	2.01 (1.14)	1.08	0.32	0.67	0.96	1.11	1.08	1.05
My heart races or palpitates when I think about getting COVID-19	2.35 (1.22)	0.54	− 0.80	0.79	1.21	0.42	0.87	0.84

MnSq = mean square

Table 2 also shows the IRT results of the FCV-19S. More specifically, all the infit and outfit MnSq values were between 0.5 and 1.5: infit MnSq between 0.87 and 1.08 with outfit MnSq between 0.84 and 1.10 for the FCV-19S. The acceptable MnSq values indicate that all the items were embedded within the same latent construct. Therefore, the unidimensionality of the FCV-19S was verified (highlighting IRT’s advantage of being able to check the validity of the item and the unidimensionality of the entire instrument).

The item difficulty coefficients were between − 0.93 and 1.11 for the FCV-19S. The item discrimination coefficients were between 0.83 and 1.21 for the FCV-19S. Apart from the item information on the item difficulties and item discriminations, the results of IRT indicate that the participants’ ability coefficients in responding to FCV-19S were between 5.60 and 6.26 (for detailed information). Therefore, the item difficulties presented in Table 2 were not influenced by the participants’ abilities (highlighting IRT’s advantage of being able to separately evaluate the ability of the individual and the difficulty of the item). In brief, this finding indicated that each item on the scale exhibited a satisfactory response in terms of difficulty as well as discrimination between the participants.

The Likert-type scale used in the FCV-19S was supported by the proper threshold ordering as outlined in Table 3. Both average and step measures were monotonically increased, and both infit and outfit MnSq values were between 0.5 and 1.5. Therefore, the descriptors used in the FCV-19S were in monotonical order, and these descriptors were appropriate (highlighting IRT’s advantage of being able to examine the appropriateness of the descriptors for the response).

**Table 3:** Threshold disordering tests for the Fear of COVID-19 Scale (FCV-19S)

	Average measure	Step measure	Infit MnSq	Outfit MnSq
1	− 2.60	-	0.92	0.95
2	− 1.37	− 2.23	0.94	0.91
3	− 0.32	− 0.66	0.90	0.88
4	0.55	0.02	1.08	1.12
5	1.87	2.88	1.27	1.24

MnSq = mean square

Additionally, no DIF contrast had an absolute value larger than 0.5 (Table 4) concerning the following characteristics: gender (male vs. female), age (less than 25.56 years vs. older than 25.56 years), living status (single vs. married), and education (having a diploma or lower education vs. having a university education). This indicates that participants interpreted the FCV-19S items in the same way in relation to these characteristics. However, a substantial DIF was displayed for six FCV-19S items (Items 1, 2, 3, 5, 6, and 7) regarding health status (self-reported poor health vs*.* self-reported acceptable or good health) and one item (Item 3) regarding residence (living in rural area vs. living in urban area). These findings suggest that FCV-19 items should not be used to compare fear of COVID-19 among individuals with different self-reported health status and those residing in different residences.

**Table 4:** Test for differential item functioning (DIF) for the Fear of COVID-19 Scale (FCV-19S)

Item and and description	DIF contrast across gender ^a,b^	DIF contrast across age ^a,c^	DIF contrast across living status ^a,d^	DIF contrast across education status ^a,e^	DIF contrast across residence status ^a,f^	DIF contrast across health status ^a,g^
**FCV-19S**						
I am most afraid of COVID-19	− 0.08	0.00	− 0.12	0.27	0.28	0.72
It makes me uncomfortable to think about COVID-19	0.13	0.09	0.11	0.29	0.22	0.59
My hands become clammy when I think about COVID-19	− 0.24	− 0.22	− 0.03	− 0.30	− 0.57	− 0.52
I am afraid of losing my life because of COVID-19	0.13	− 0.25	− 0.31	0.0	0.28	0.11
When watching news and stories about COVID-19 on social media, I become nervous or anxious	0.03	0.00	0.11	0.17	0.10	0.54
I cannot sleep because I’m worrying about getting COVID-19	− 0.19	0.31	0.26	− 0.16	− 0.16	− 0.85
My heart races or palpitates when I think about getting COVID-19	0.15	0.09	0.02	− 0.31	− 0.35	− 0.63

^a^DIF contrast > 0.5 indicates substantial DIF

^b^DIF contrast across gender = Difficulty for males-Difficulty for females

^c^DIF contrast across age groups = Difficulty for younger (i.e., ≤ 25.56 years)-Difficulty for older (i.e., > 25.56 years) people

^d^DIF contrast across living groups = Difficulty for single (living alone) -Difficulty for married people

^e^DIF contrast across education groups = Difficulty for people with diploma or lower education -Difficulty for people with university education

^f^DIF contrast across residence groups = Difficulty for people who are living in rural areas -Difficulty people who are living in urban areas

^g^DIF contrast across health status groups = Difficulty for people who reported their health status as poor -Difficulty for people who reported their health status as acceptable or good

*MnSq* mean square error, *DIF* differential item functioning

**Fig. 1:**
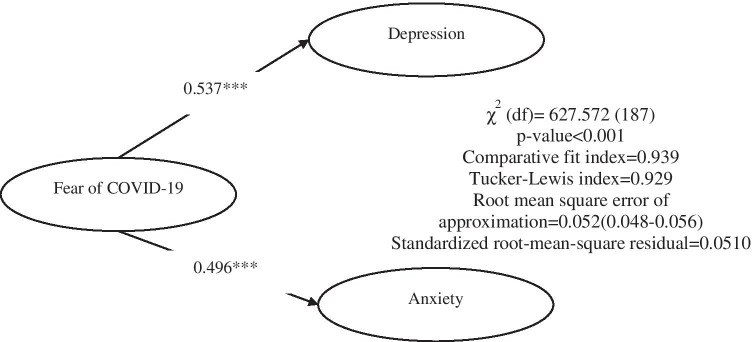
Concurrent validity of the Fear of COVID-19 Scale (FCV-19S) with the external criteria of anxiety and depression. *** *p* < .001

Concurrent validity of the FCV-19S was examined by examining correlations between the FCV-19S using SEM (Fig. 1). The latent score of the FCV-19S scale was significantly and positively associated with the two latent scores of the HADS subscales (anxiety: *β* = 0.537, *p* < 0.001; depression: *β* = 0.496, *p* < 0.001).

## Discussion

Psychological distress has been commonly used as a mental health indicator (Deasy et al., 2014), and depression and anxiety symptoms are commonly viewed as being forms of psychological distress. Psychological distress comprises a far broader spectrum of conditions than mental illness, from minor symptoms to serious psychological disorder (McLachlan & Gale, 2018). During the COVID-19 pandemic, the focus has arguably been more on prevention and treatment of the disease. However, it is just as important to be concerned about the mental health aspects, which can only be evaluated using reliable assessment tools.

In the present study, the main aim was to assess the reliability and validity of the Urdu version of the recently developed Fear of COVID-19 Scale (FCV-19S). The internal consistency of the scale (using Cronbach’s α) was shown to be very good (α = 0.88), which was similar to that reported in other validated versions of the FCV-19S including those in Turkish (0.85; Satici et al., 2020), Persian (0.82; Ahorsu et al., 2020), Bangla (0.87; Sakib et al., 2020), Arabic (0.88; Alyami et al., 2020), Russian (0.81; Gritsenko et al., 2020), Italian (0.87; Soraci et al., 2020), and Hebrew (0.86; Bitan et al., 2020). The unidimensionality of the scale was confirmed by CFA, and the factor loadings were found to be satisfactory, indicating a good construct of the scale. Similar findings were reported by other validation studies (e.g., Alyami et al., 2020; Haktanir et al., 2020; Satici et al., 2020; Soraci et al., 2020).

Many individuals fear being infected with COVID-19 because of relatively high risk of causing death (Kobayashi et al., 2020). Strict quarantine measures have caused alarm among the public because they are likely to cause financial instability, irritability, and boredom (Brooks et al., 2020). Individuals with confirmed or suspected COVID-19 are at a risk of suffering from fear from severe disease consequences or infecting others (Dsouza et al., 2020). Among frontline healthcare workers, excessive workload, isolation, and discrimination have led to exhaustion as well as fear and sleep disturbances (Kang et al., 2020). All of the aforementioned factors can lead to stress, anxiety, and/or depression (Brooks et al., 2020). According to a recent study conducted in China, the overall prevalence of anxiety and depressive symptoms among the general public were 35.1% and 20.1%, respectively (Huang and Zhao 2020).

After analyzing the factorial properties of the scale, Rasch analysis was used to examine the validity and characteristics of each individual item on the FCV-19S. This was used because IRT models yield item and latent trait estimates, which are not affected by the (i) characteristics of the population with respect to the underlying trait, (ii) standard errors depending on trait level, or (iii) trait estimates related to item content (Hays et al. 2000). Furthermore, concurrent validity analysis (using the latent variable modeling in SEM) showed that the FCV-19S has significant positive correlations with both subscales of the HADS (and more so with anxiety). Similar findings were also reported in other FCV-19S validation studies (e.g., Ahorsu et al., 2020; Alyami et al., 2020; Sakib et al., 2020; Satici et al., 2020; Soraci et al., 2020). According to a study by Chew et al. (2020), fear, anxiety, and depression were the most common psychological symptoms reported among virus outbreaks globally. This highly increases the likelihood of the three conditions occurring concurrently.

Overall, the findings of the present study were similar to other published studies. For the psychosocial care of every individual, further studies involving a larger subset of the population should be carried out to provide more detailed insight concerning the psychosocial impact of fear, anxiety, and depression symptoms in relation to other behaviors such as hand washing, adhering to quarantining, spatial distancing measures, social media use, etc.

A few limitations should be noted when interpreting the findings. The present study was carried out when strict quarantine measures were in place in Pakistan and meant that recruitment of participants used a convenience sampling technique. Moreover, the data were collected via social media and therefore the present sample might be socio-culturally limited (i.e., those without the access to internet might not be recruited and this group may be more economically disadvantaged). This may have caused a selection bias because the sample cannot be generalized to the Pakistani population. The study’s data were all self-report in nature and are subject to well-established methods biases. Despite these limitations, this study demonstrates that the Urdu version of the FCV-19S is a valid seven-item unidimensional scale with robust psychometric properties and is a useful tool to assess fear of COVID-19 among the Pakistani adults.
